# No Such Thing as Chronic Q Fever

**DOI:** 10.3201/eid2305.151159

**Published:** 2017-05

**Authors:** Matthieu Million, Didier Raoult

**Affiliations:** French National Referral Center for Q Fever, Marseille, France

**Keywords:** Coxiella burnetii, Q fever, chronic Q Fever, persistent focalized Coxiella burnetii infection, endocarditis, cardiovascular infections, systematic echocardiography, valvulopathy, bacteria, treatment, antimicrobial drug prophylaxis, the Netherlands, France

## Abstract

Modern diagnostic methods enable clinicians to look beyond a diagnosis of chronic Q fever and discern whether patients instead have persistent focalized *Coxiella burnetii* infection(s). Use of these methods and development of criteria to define and treat such infections, especially cardiovascular infections, will improve the prognosis for patients previously thought to have chronic Q fever.

We read with interest the article by Kampschreur et al. (*1*), which in our opinion conveys a perspective that is incorrect concerning the diagnostic algorithm and treatment of Q fever. Kampschreur et al.’s article characterizes the understanding and management of Q fever by the Dutch Q Fever Consensus Group. However, this consensus opinion may be erroneous if developed without input from disease experts with long clinical experience.

Kampschreur et al.’s use of the term chronic Q fever is misleading because it may lead to inadequate treatment of persistent focalized *Coxiella burnetii* infection(s). The obsolete term chronic Q fever should be abandoned to prevent confusion between endocarditis, vascular infections, osteoarticular infections, lymphadenitis, genital infection, and pericarditis, which occurred in 68%, 20%, 7%, 6%, 3%, and 1%, respectively, of 494 patients with persistent focalized infection(s) who we followed during 2007–2015 at the French National Referral Center for Q Fever in Marseille, France (unpub. data).

Another example of the deleterious effect of the Dutch Q Fever Consensus Group guidelines is the absence of screening echocardiography in the standard work-up for patients with Q fever in the Netherlands (*2*). Because endocarditis has been reported in patients with clinically silent, undiagnosed valvulopathies (*3*), we recommend systematic echocardiography for all persons with acute Q fever. Endocarditis develops in most untreated Q fever patients who have extensive valvulopathy; however, Million et al. (*4*) showed that it did not develop in patients who received prophylaxis. This finding led us to recommend prophylaxis for acute Q fever patients with valvulopathy at the French National Referral Center for Q Fever; over the past 10 years, this strategy has reduced the incidence of Q fever endocarditis in patients at the center (*5*). Despite these observations, which were confirmed in the Netherlands in 2015 (*6*), the standard work-up for Q fever patients in that country has not included screening echocardiography since 2010 (*2*), leaving patients with clinically silent valvulopathy untreated.

Specific defining criteria for endocarditis (*7*) are needed to enable comparison of clinical series. Use of the term chronic to define cardiovascular infections in patients with Q fever is misleading. Indeed, valvular vegetations were recently reported in acute Q fever (*8*). Q fever vascular infections must be distinguished in the context of mycotic aneurisms, small saccular and embolic consequences of endocarditis that may go unnoticed, and underlying vascular disease. Positron emission tomography (PET) scanning has been used effectively in the Netherlands to systematically detect the localization of infection in patients with elevated serologic test results (*9*). PET scanning dramatically improves the diagnosis of cardiovascular infections (*10*). However, because the Dutch criteria lack clinical relevance (*7*), many cases of endocarditis were missed, and diagnoses of vascular infection were retained in the presence of mycotic aneurysms. These misdiagnoses explain the low proportion of endocarditis cases compared with vascular infections in the Dutch series (15% vs. 36%, as reported by Kampschreur et al. (*1*)) compared with the series in our center (68% vs. 20%; unpub. data).

Endocarditis and vascular infections, whose first symptoms may be fatal decompensation or stroke, can be prevented in Q fever patients by implementing systematic screening echocardiography, phase I IgG monitoring, and PET scanning of patients with vascular disease (*10*). In our experience, only 1 patient with uncontrolled Q fever endocarditis has died since 2006, when we began following this protocol (*3*). The patient had a cardiac valve replacement 1 year before dying, but his phase I IgG titer was low (1:200), and *C. burnetii* PCR for his valve was negative, so no treatment was prescribed.

Reanalysis of the Q fever literature by different teams has brought challenging concepts to light (*7*). In a series from the Netherlands (*1*), 4 patients were shown to have died from endocarditis and 2 from vascular infections. These patients may have had better outcomes if the methods we propose here had been followed. Conversely, high serologic titers are not definite proof of persistent focalized infection, as illustrated in an outbreak in French Guiana, where exceptionally high serologic titers have been observed, but persistent focalized infections have rarely been diagnosed (*10*).

Accurate identification of persistent focalized *C. burnetii* infections will improve patient outcomes by preventing long-term, organ-specific, lethal complications (e.g., vascular infections are a risk for vascular rupture, lymphadenitis is a risk for lymphoma) and by avoiding drug side effects in patients with isolated elevated serologic test results. Clinicians should look beyond a diagnosis of chronic Q fever to determine whether a patient might have persistent focalized infection(s). The term fever in Q fever has evolved from a pathologic picture per se to a clinical epiphenomenon; it is now time to evolve from the concept of chronic Q fever to one of persistent focalized *C. burnetii* infection(s) (*10*).
